# Immediate effects of urgent reorganisation of emergency department-based treatment pathway in nonperforated appendicitis: a retrospective study

**DOI:** 10.1186/s12873-020-00339-6

**Published:** 2020-05-29

**Authors:** Artur Ojakäär, Martin Purdy, Aristotelis Kechagias, Ulla Järvelin, Ari Palomäki

**Affiliations:** 1 Department of Surgery, Kanta-Häme Central Hospital, Ahvenistontie 20, FI-13530 Hämeenlinna, Finland; 2 Department of Emergency Medicine, Kanta-Häme Central Hospital, Ahvenistontie 20, FI-13530 Hämeenlinna, Finland; 3 Department of Surgery, Rea Hospital, Siggrou Avenue 383, 175 64 P. Faliro, Athens, Greece; 4 Department of Emergency Medicine Acuta, Tampere University Hospital, Teiskontie 35, FI-33520 Tampere, Finland; 5 Faculty of Medicine and Health Technology, Tampere University, Kauppi Campus, Arvo Ylpön katu 34, FI-33520 Tampere, Finland

**Keywords:** Appendicectomy, Appendectomy, Day surgery, Reorganisation, Emergency department

## Abstract

**Background:**

Acute appendicitis is a global disease and a very common indication for emergency surgery worldwide. The need for hospital resources is therefore constantly high. The administration in Kanta-Häme Central Hospital, Southern Finland, called for an urgent reorganisation due to shortage of hospital beds at the department of general surgery. Postoperative treatment pathway of patients with nonperforated acute appendicitis was ordered to take place in the Emergency Department (ED). The aim of this study was to assess, whether this reorganisation was feasible and safe, i.e. did it affect the length of in-hospital stay (LOS) and the 30-day complication rate.

**Methods:**

This is a retrospective pre- and post-intervention analysis. After the reorganisation, most patients with nonperforated appendicitis were followed postoperatively at the 24-h observation unit of the ED instead of surgical ward. Patients operated during the first 3 months after the reorganisation were compared to those operated during the 3 months before it. A case met inclusion criteria if there were no signs of appendiceal perforation during surgery. Exclusion criteria comprised age < 18 years and perforated disease.

**Results:**

Appendicectomy was performed on 112 patients, of whom 62 were adults with nonperforated appendicitis. Twenty-seven of the included patients were treated before the reorganisation, and 35 after it. Twenty of the latter were followed only at the ED. Postoperative LOS decreased significantly after the reorganisation. Median postoperative time till discharge was 15.7 h for all patients after the reorganisation compared to 24.4 h before the reorganisation (standard error 6.2 h, 95% confidence interval 2.3–15.2 h, *p* < 0.01). There were no more complications in the group treated postoperatively in the ED.

**Conclusions:**

Early discharge of patients with nonperforated appendicitis after enforced urgent reorganisation of the treatment pathway in the ED observation unit is safe and feasible. Shifting the postoperative monitoring and the discharge policy of such patients to the ED – instead of the surgical ward – occurred in the majority of the cases after the reorganisation. This change may spare resources as in our series it resulted in a significantly shorter LOS without any increase in the 30-day complication rate.

## Background

Discharge time after operation of nonperforated appendicitis has been significantly shortened in the last 35 years. In the 1980s there was an average discharge at 3–4 days postoperatively [1]. Since the 1990s, it has been a standard practice to discharge patients within 24–48 h [2, 3]. The development of laparoscopic surgery further improved early discharge rates after appendicectomy without additional complications [4].

Kanta-Häme Central Hospital (K-HCH) is a secondary care hospital located at South of Finland. The Emergency Department (ED) of K-HCH has modern facilities and a subunit dedicated to the short-term (≤ 24 h) observation of patients with an acute condition [5]. In addition, emergency medicine has been introduced as a separate specialty branch in Finland in 2013 [6]. Since then, there has been a continuous reappraisal and development of its functionality within the inter-disciplinary management of patients, such as those operated for an acute abdominal condition [7–10].

A recent administrative decision of K-HCH called for an active participation of the ED in the in-hospital management of adult patients with acute appendicitis. The intent was to reduce the use of hospital beds at the department of general surgery within a few weeks. These enforced, rapid changes led to a reorganisation according to which the patients with nonperforated appendicitis would be followed preferentially at the ED during the pre- and postoperative period. For this reason, a new protocol for a same-day patient discharge was implemented in the facilities of the ED without delay.

The objective of this retrospective study is to analyse the immediate effect of this new obliged protocol on the length of stay (LOS) of adult patients operated for nonperforated acute appendicitis and on the 30-day complication rate. Our special interest is to assess whether the reorganisation is feasible, i.e. could we safely shorten LOS when patients were treated pre- and postoperatively at the ED instead of the surgical ward.

## Methods

We conducted a retrospective study on all adult patients undergoing appendicectomy for suspected acute appendicitis during a six-month period: 3 months before and 3 months after the reorganisation. Data were gathered from the electronic patient record system and from the operation registry of the K-HCH. Patients with the Nordic Medico-Statistical Committee (NOMESCO) code JEA00 or JEA01 for open and for laparoscopic appendicectomy were included. Clinical suspicion or radiological diagnosis of acute appendicitis were the indications for surgery.

We included patients at least 18 years old. Patients with normal appendix were excluded from the analysis. An intra-operative scoring system was used to grade the severity of appendicitis (Table 1) [11]. Patients with grade Ia to Ic appendicitis without perforation or any other significant complications were included in the analysis.

**Table 1:** Intra-operative scoring of appendicitis [11]

Grade	
0	No appendicitis
Ia	Oedematous, ingurgitated appendix
Ib	Abscessed or phlegmonous appendix, presents fibrin membranes and seropurulent liquid around appendix
Ic	Necrosed appendix with no perforation
II	Perforated appendix with localized abscess
III	Complicated appendicitis with generalized peritonitis

We retrospectively studied the postoperative LOS of patients with acute nonperforated appendicitis. The results over the three-month periods immediately before and after the reorganisation were compared to each other. Before the reorganisation, patients were always followed at the department of surgery, which included at least one clinical visit from a surgical consultant or resident on the first postoperative morning. According to the reorganisation protocol, a patient operated on nonperforated disease would be preferentially followed at the ED with an intent of same-day discharge in case of an uneventful postoperative course. Our ED has per se the flexibility of a broader discharge-timetable (from 8:00 to 00:00) compared to the surgical ward discharge policy, which is during office hours with a short additional time frame. In order not to compromise safety after the reorganisation, the final decision for a postoperative triage towards the ED or the surgical ward remained at the clinical judgement of the operating surgeon. The operative report depicted precise postoperative instructions and surgical consultation was readily available if needed. The ED nurses had the duty for the continuous assessment of the patient’s clinical status postoperatively, and the doctor on-call (in most cases an early-career ED resident) had the responsibility of the discharge. Therefore, the reorganisation shifted the discharge process to the ED after surgery for nonperforated appendicitis, with surgical consultation only on demand.

There was no change in the pre-operative nor the intraoperative modalities due to the reorganisation. For example, the triage of the patient and the timing from admission to surgery, the anaesthesia protocol and the surgical techniques remained the same. At the time of this study, anaesthetic induction was achieved with the use of fentanyl and rocuronium bromide at a dose of 0,5 mg/kg. All patients were reversed with neostigmine or sugammadex according to the measurements of the neurostimulation. Local wound anaesthesia was routinely administered. Paracetamol and ibuprofen were routinely used as the postoperative pain medication. Short-acting opioids were used only in the uncommon occasion in case the aforementioned scheme would not suffice.

We documented complications and readmissions for 30 days postoperatively. Patients receiving any operation at our institution are referred to us for any complication that cannot be managed at the level of the general practitioner, according to a strict referral system. The demographics of the patients treated before and after the reorganisation of the process were compared to each other. Moreover, we analysed time of presentation (during office hours vs out-of-office hours), physical status based on the classification of the American Society of Anesthesiologists (ASA) and surgical technique, i.e. laparoscopic vs open surgery.

## Statistical methods

We performed statistical analyses using IBM® SPSS® Statistics Version 24 (copyright 2016). Data are presented as medians (minimum–maximum, interquartile range) in cases of non-normality. We made a priori a sample size calculation for postoperative LOS (SD 40%), with the presumed sampling ratio of 5:6. For the effect of 35% (α = .05, 2 tailed), we would have needed approximately 22 patients in the group 2 to yield power of 80%. We assessed differences in continuous variables using the Mann–Whitney *U*-test. Differences in dichotomous variables were analysed using the Chi- square test or Fischer’s exact test as appropriate. A probability value < 0.05 was considered significant.

## Results

**Fig. 1:**
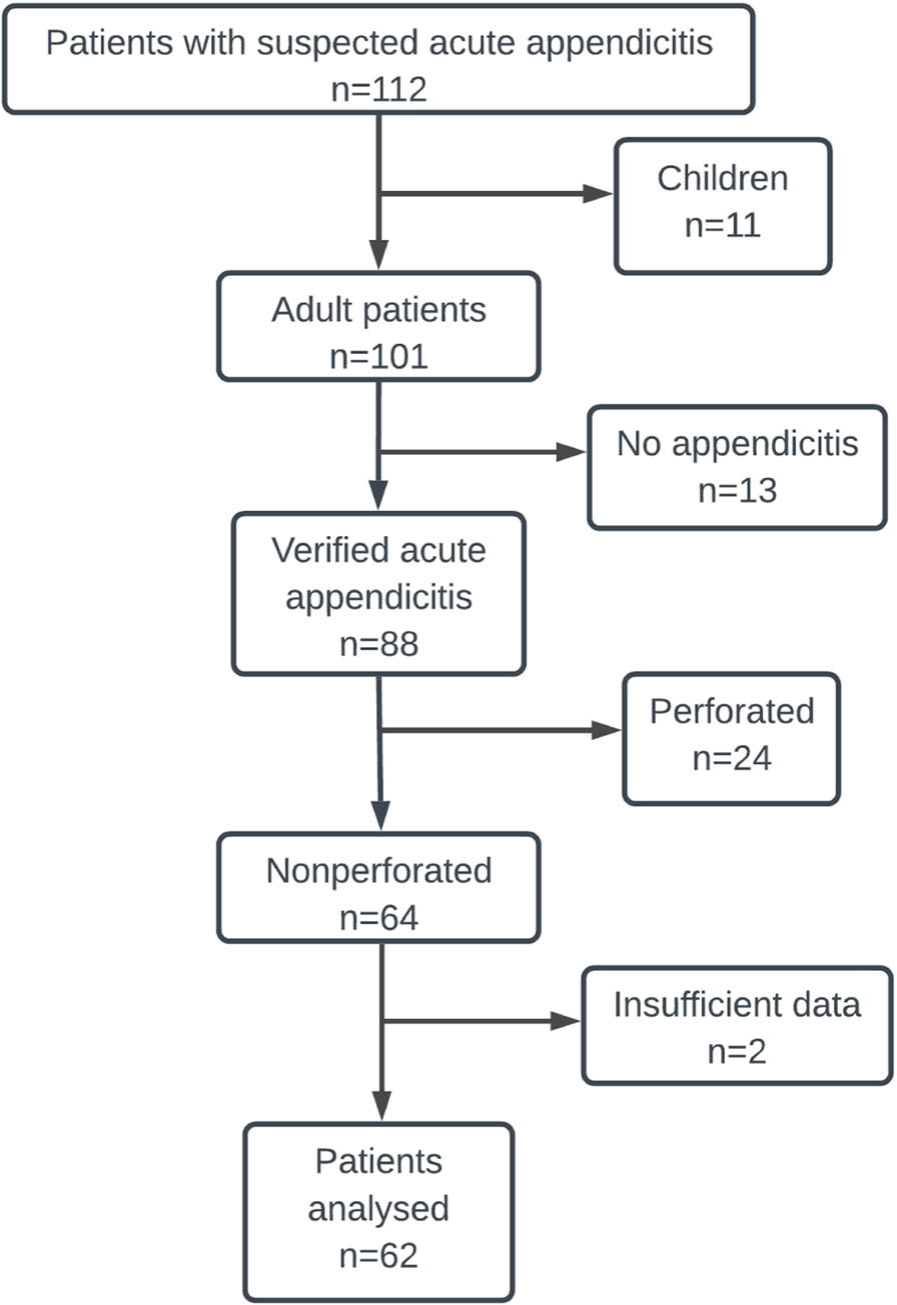
Flowchart of the study

During the study period, a total of 112 patients with acute appendicitis were operated on. Sixty-two (61.4%) adult patients with nonperforated appendicitis were included in the analysis. Figure 1 presents detailed information on inclusion and exclusion criteria.

Of the patients evaluated, 27 were women and 35 men. The difference in gender between the groups treated before and after the reorganisation of our process was not significant. Median age of patients was 33 years (min – max: 18–78) with no significant differences between the groups (NS). Patients’ characteristics are presented in Table 2. Further, there were neither any significant differences concerning BMI, ASA class or use of laparoscopic operation technique. Patients were also admitted to the hospital during office-hours similarly before and after the reorganisation (Table 2).

**Table 2:** Patients’ demographics and characteristics before and after the reorganisation of the treatment process

Variable	Before	After	*p* value
Number of patients	27	35	
Age, median	29	36	N.S.
Gender (men / women)	9 / 18	18 / 17	N.S.
Body mass index, mean (SD)	26.2 (4.9)	25.9 (5.5)	N.S.
Presentation during office hours^a^	13	15	N.S.
ASA classification 1–2/3	24 / 3	31 / 4	N.S.
Laparoscopic surgery	19	25	N.S.

^a^Office hours: Monday to Friday, 8 AM–4 PM

**Fig. 2:**
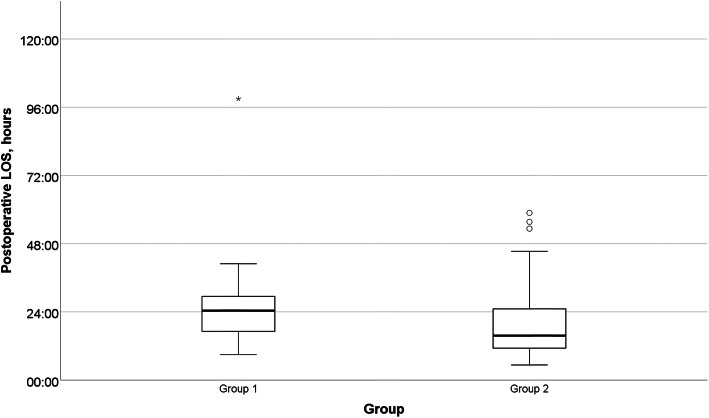
Postoperative length-of-stay before and after the reorganisation

Before the reorganisation, all of 27 patients (100%, Group 1) were followed postoperatively at the surgical ward. Immediately after the start of the reorganisation (Group 2), 20 patients (57.1%) were followed at the ED observation unit and 15 (42.9%) at the surgical ward. The results concerning postoperative stay in hospital are presented in Fig. 2. The median postoperative LOS decreased significantly by 46.2%, being 24.4 h and 15.7 h before and after the reorganisation, respectively (*p* = 0.023), with no significant changes in the time from admission to surgery (10.3 h vs 10.0 h, *p* = 0.865) nor the duration of the operation (49 min vs 54 min, *p* = 0.356). When only patients in Group 2 (after the reorganisation) were compared, those treated postoperatively at the surgical ward and at the ED-only had a median postoperative LOS of 25.0 h and 13.1 h (*p* = 0.025), whereas the duration of operation was 67 min vs 45.5 min (*p* = 0.014) and the time from hospital admission to surgery was 10.9 h vs 9.9 h (*p* = 0.633), respectively. The comparison between patients treated postoperatively in the ED with all the patients treated postoperatively in the surgical ward before and after the reorganisation showed no differences in the interval between hospital admission to surgery (9.9 h vs 10.4 h, *p* = 0.758) and in the duration of the operation (45.5 min vs 58 min, *p* = 0.157), whereas the median postoperative LOS decreased significantly (13.13 h vs 24.73 h, *p* = 0.001).

All the patients before the reorganisation were discharged from the surgical ward between 11:30 and 17:37. The discharge for Group 2 patients from the ward and from the ED occurred between 10:08 and 15:49 and between 08:09 and 23:24, respectively. The ASA classification of 20 patients discharged from the ED was as follows: 14 patients in ASA 1, five in ASA 2 and one in ASA 3. One patient in Group 2 who was treated postoperatively on the surgical ward had a superficial wound infection at 30-day follow-up (NS). All other patients in Groups 1 and 2 remained free from postoperative complications. There were no readmissions.

## Discussion

In this study, we evaluated the length of postoperative hospital stay in patients operated on for nonperforated acute appendicitis after a reorganisation oriented to same-day discharge. We showed that although the administratively enforced reorganisation was determined and executed in a short period of time, our immediate results were good. LOS was significantly shorter in patients treated at the ED than among those treated at the surgical ward postoperatively. The postoperative direction of the patient to the ED was successful in the majority of the cases after the reorganisation, despite the fact that the time to implement the new protocol for patient management was only a few weeks. This corresponds to a rather satisfactory initial compliance of the medical and nursery staff in the implementation of the new protocol. We also calculated a shorter operative time in patients who were observed in the ED compared to the patients who were directed to the surgical ward after the reorganisation. This finding implies that the operating surgeon’s clinical judgement remained the major determinant of maintaining safety concerning the postoperative triage.

Our findings concur with earlier studies, where early discharge of patients with non-perforated acute appendicitis was reported to be safe and effective [12, 13]. A review of 13 studies with 1152 adult patients who underwent day-case appendicectomy reported that only 27% were discharged within 12 h, 53% within 24 h, and 21% within 72 h [14]. The cases of our study that were followed at the ED after the reorganisation (57.1%) approached a discharge within 13 h from the operation. Thus, the majority of our Group 2 patients virtually reached the most favourable benchmark of the aforementioned review [14]. Interestingly, there are further studies from the United States and Canada that have reported impressive results with a postoperative discharge within 3–4.7 h in the majority of their series (45–86%) [12, 13]. This was associated with a return-rate to the ED of 8–11.4% (the respective rate was 1.6% in our series) without any need for in-hospital readmission [12, 13]. The Canadian study showed that the early-discharge policy resulted in a 45% reduction in the need for in-hospital beds [13]. Same-day discharge is therefore feasible and effective. Possibly the variation in the length of postoperative stay between different centres can be attributed to modifiable local factors and results can be further improved with extensive training [15]. In addition, early discharge is feasible and reliable also in paediatric patients [16].

An important aspect of the herein research is that it provides real-life results concerning early discharge after surgery for nonperforated acute appendicitis. To the best of our knowledge there is scarce evidence concerning early or same-day discharge from the Nordic Countries and particularly from Finland. It is interesting to note that the main proponent of non-operative management for nonperforated acute appendicitis, which is the APPAC multi-centre randomised trial from Finland between 2009 and 2012, did not take into account the potential benefits of early discharge to the comprehensive outcome. According to the APPAC conservative therapy with antibiotics was non-inferior to surgery [17]. At 5 years, the majority (61%) of antibiotic group patients did not undergo appendicectomy and the overall costs of the surgical arm were 1.4 times higher [18, 19]. No information was provided concerning the time from surgery to discharge [17]. Moreover, in the surgery group, only standard open appendicectomy was performed [17] and possibly this may have contributed to a delayed discharge in the APPAC population compared to modern laparoscopic appendicectomy. On the contrary, it has been shown that early discharge after appendicectomy confers a significant reduction in the costs [20]. We believe that the results of our study should be taken into account in this context, as the overall benefit of non-operative treatment for nonperforated acute appendicitis could be challenged from an aggressive early discharge policy after laparoscopic appendicectomy.

The potential weaknesses of a study with a retrospective design are contained in this series as the major end-points are electronically recorded during the routine clinical practice at our institution. The time of surgery and the time of discharge are always digitally documented on the spot. In addition, there is a valid electronic documentation of the 30-day complications due to a strict post-discharge policy that comprises direct telephone consultation from the patient’s family doctor and referral for complication management requisitely to our institution. On the other hand, there is a possibility of a selection bias given the fact that there was not a robust compliance to the reorganisation’s policy, at least during the initial 3-month period. Indeed, 15 out of the 35 patients were directed to the surgical ward instead of the ED, which could be explained in a lesser extent by the surgeon’s preference not to deviate from the previous routine [15]. However, the finding that the former patients had a longer duration of the operation possibly reflects a relatively increased operative difficulty as the reason of the surgeon’s decision for an expert supervision at the surgical ward and with the intent not to compromise safety.

Although this study is based on a relatively small series, it shows that a major change in routine practice is feasible even at a short time and at a satisfactory compliance rate, and with immediate positive outcome in respect of the initial objective, which was the safe reduction of the postoperative LOS. Another interesting feature of the herein investigation is that the responsibility of the postoperative course of patients operated readily for nonperforated acute appendicitis can be safely assigned to a non-surgical specialty such as emergency medicine, and even at the level of doctors in training, with the provision of precise postoperative instructions by the surgeon and the readiness for a surgical consultation. In addition, the reorganisation exploited the inherent feature of the ED to discharge a patient over a wider time frame in order to reduce decisively the postoperative LOS. It is also possible that further reorganisation of the whole in-hospital management, and particularly a more active role of the recovery room to the early discharge, could result in an even shorter LOS than the one documented in this study [20, 21]. Last, this study confirmed that surgery for nonperforated appendicitis bears minimal postoperative morbidity as there was not any significant complication in all the series. Therefore, despite its moderate sample-size, our study is in favour, that early discharge does not affect postoperative complication and readmission rate.

## Conclusions

This retrospective analysis extends over the period during which we had to reorganise whole postoperative treatment of patients with acute nonperforated appendicitis in the ED in a very limited time. In this study, postoperative LOS was significantly shorter after the reorganisation than before it. This reflects the feasibility and the positive impact of the implementation of a same-day discharge protocol. Moreover, shifting the discharge process and responsibility to a non-surgical unit such as the ED, proved to be functional and safe.

## Data Availability

Not applicable.

## Abbreviations

ASA: American Society of Anesthesiologists
BMI: Body mass index
ED: Emergency Department
K-HCH: Kanta-Häme Central Hospital
LOS: Length of stay
NOMESCO: Nordic Medico-Statistical Committee
